# Kinematic and Electrophysiological Characteristics of Pedal Operation by Elderly Drivers during Emergency Braking

**DOI:** 10.3390/healthcare9070852

**Published:** 2021-07-06

**Authors:** Kazuki Fujita, Yasutaka Kobayashi, Mamiko Sato, Hideaki Hori, Ryo Sakai, Tomoki Ogawa, Tomonari Sugano, Kaori Kawabata, Masahito Hitosugi

**Affiliations:** 1 Graduate School of Health Science, Fukui Health Science University, Fukui 910-3190, Japan; yasutaka_k@fukui-hsu.ac.jp (Y.K.); rpt-hori@fukui-hsu.ac.jp (H.H.); kawabata-ot@fukui-hsu.ac.jp (K.K.); 2 Department of Rehabilitation Medicine, Fukui General Hospital, Fukui 910-8561, Japan; satomoko@f-gh.jp; 3 Department of Rehabilitation, Faculty of Health Science, Fukui Health Science University, Fukui 910-3190, Japan; r-sakai@fukui-hsu.ac.jp (R.S.); t.sugano@fukui-hsu.ac.jp (T.S.); 4 Department of Physical Therapy Rehabilitation, Fukui General Hospital, Fukui 910-8561, Japan; fsmsc@f-gh.jp; 5 Department of Legal Medicine, Shiga University of Medical Science, Otsu 520-2192, Japan; hitosugi@belle.shiga-med.ac.jp

**Keywords:** motor vehicle collisions, car driving, pedal error, electromyography, motor control

## Abstract

Age-related decline in lower limb motor control may cause errors in pedal operation when driving a car. This study aimed to clarify the kinematics and electrophysiological characteristics of the pedal-switching operation associated with emergency braking in the case of elderly drivers. The participants in this study consisted of 11 young drivers and 10 elderly drivers. An experimental pedal was used, and the muscle activity and kinematic data during braking action were analyzed using the light from a light-emitting diode installed in the front as a trigger. The results showed that elderly drivers took the same time from viewing the visual stimulus to releasing the accelerator pedal as younger drivers, but took longer to switch to the brake pedal. The elderly drivers had higher soleus muscle activity throughout the process, from accelerator release to brake contact; furthermore, the rectus femoris activity was delayed, and the simultaneous activity between the rectus femoris and biceps femoris was low. Furthermore, elderly drivers tended to have low hip adduction velocity and tended to switch pedals by hip internal rotation. Thus, the alteration in joint movements and muscle activity of elderly drivers can reduce their pedal operability and may be related to the occurrence of pedal errors.

## 1. Introduction

A study of the major causes of fatal motor vehicle collisions (MVCs) in Japan in 2018 showed that for drivers under the age of 75 years, the major cause of MVCs was a lack of attention in the driving direction and unconfirmed safety, whereas for drivers older than 75 years, improper operation was the major cause [1]. In particular, the percentage of fatal MVCs due to miss-stepping between the brake and accelerator (i.e., pedal error) was 0.6% for those under 75 years old, but a high value of 7.8% was found for those over 75 years old [1]. That is, pedal errors by elderly drivers cause serious collisions such as those arising from the vehicle being out of control, which is a significant problem in Japan.

The American Medical Association guidelines state that driving a car safely requires the following three functional elements: vision, cognition, and motor functions [2]. In Japan, elderly drivers over 75 years of age are required to undergo a cognitive function test in addition to a vision test when renewing their license. Hence, there should be no significant abnormality in the cognitive function of those who had the pedal errors. As switching from the accelerator to the brake pedal of a car is a simple action, the influence of cognitive function is regarded as being small. In fact, trail making test and mini-mental state examination (MMSE) were reported not to be associated with pedal errors [3]. Pedal errors may be more affected by age-related decline in motor function than in cognitive function. For example, age-related weakness of the lower limb muscles [4] and loss of ability to develop ankle-joint torques rapidly [5] may affect the braking action, but these alone are unlikely to cause pedal errors. Pedal errors are characterized by back-pedal hooks (foot catching the underside or side of the brake pedal when transitioning from accelerator), incorrect trajectories, uncertain foot wags, misses, slips, and other behaviors [6]. To understand this pedal error problem, Cantin et al. [7] analyzed the linear displacement of the right foot during braking action in elderly drivers, and reported that the movement variability was large. In addition, Lodha et al. [8] showed that the ankle motor output varied greatly during braking action in elderly drivers. Therefore, age-related decline in foot motor control may be associated with pedal errors [7,8]. However, the neurophysiological features related to motor control during emergency braking in elderly drivers are unknown.

In the case of young drivers, the electromyograms of the ankle dorsiflexors and plantar flexors during emergency braking have been measured in previous studies [9,10,11]. The tibialis anterior and plantar flexors play important roles in controlling the force exerted by the foot on the accelerator while driving at a constant speed [10]. Then, in the event of an urgent need for braking, the tibialis anterior muscles become more active in the early stage to quickly release the sole from the accelerator pedal [12]. When the activity of the ankle dorsiflexor increases in this way, the activity of the ankle plantar flexor as the antagonist decreases, owing to reciprocity inhibition at the spinal-cord level [13]. In particular, the stretch reflex of the ankle plantar flexors should be strongly suppressed during rapid dorsiflexion movements [13]. In fact, the onset of the triceps surae activity is delayed compared with the activity of the tibialis anterior when switching pedals for emergency braking [9]. However, reciprocity inhibition at the spinal-cord level decreases with age [14]. If this decrease affects emergency braking, the activity of the plantar flexors may not be suppressed and rapid dorsiflexion may be difficult.

Furthermore, during pedal switching for the braking action, the movement of turning the toes to the left and sliding the heel position to the left are observed [15]. At this time, the right hip joint undergoes the internal rotation and adduction movements. In addition, as the accelerator and brake pedal have different heights, flexion of the hip and knee joints is considered necessary for pedal switching. Most of the previous studies on braking action focused only on the ankle movements and lower-leg electromyograms possibly, because it was difficult to irradiate the entire lower limbs with the infrared camera of a three-dimensional motion analysis device owing to obstacles such as the car seat and steering. The study by Behr et al. [16] is perhaps the only one in which the sagittal joint angle and muscle activity of the entire lower limb in the accelerator and brake positions were measured; however, the data for pedal switching were not reported.

Elucidation of the cause of pedal error is an urgent issue to prevent serious MVCs. Many points related to kinematics and electrophysiology involved in the braking action are unclear. These points can be clarified by a three-dimensional motion analysis using an inertial sensor and a wireless device for electromyography (EMG). This study aimed to clarify the kinematics and electrophysiological characteristics of the pedal-switching operation associated with emergency braking in the case of elderly drivers.

## 2. Materials and Methods

### 2.1. Subjects

Between August 2020 and February 2021, subjects were recruited through the study guide leaflet with eligibility criteria. In total, 11 young (aged 22–39 years old) and 10 elderly (aged > 75 years old) persons participated in the study (Table 1). The inclusion criteria were as follows: (i) the subject should fall in either of the age groups, namely, 22–39 years or over 75 years; (ii) the subject should drive a car at least thrice a week; (iii) the subject should have obtained a driver’s license more than 3 years ago; (iv) the subject should be able to walk independently; and (v) the range of motion of the lower limb joints is in the normal range.

The exclusion criteria were as follows: (i) an MMSE score of <24 points [17], (ii) a history of lower limb-joint surgery, (iii) a history of neurological disorders (central nervous system disorders such as stroke or disorders that cause peripheral nerve symptoms), and (iv) ongoing treatment for cardiovascular disease. All of the subjects provided written informed consent for participation. This study was approved by the Ethical Review Committee of Nittazuka Medical Welfare Center (approval no. Nittazuka Ethics 2020-8).

### 2.2. Experimental Setup and Protocol

**Figure 1:**
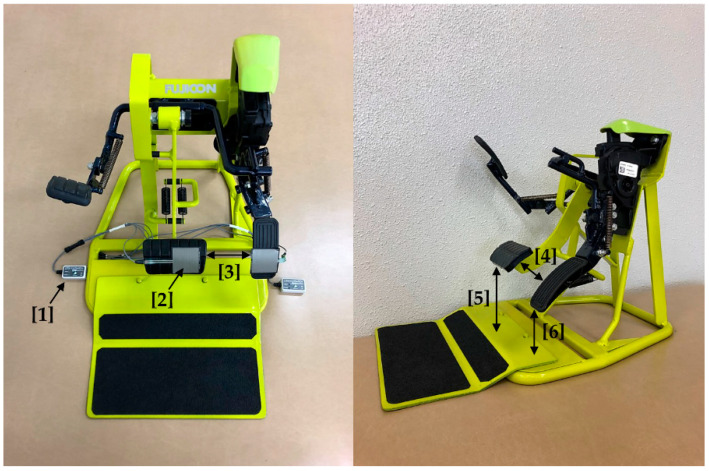
Pedals used in the experiment: [1] foot switch; [2] pressure sensor; [3] distance between the pedals of 73 mm; [4] height difference between the pedals of 30 mm; [5] brake height of 110 mm; [6] accelerator height of 75 mm.

Experimental car pedals (Fujiauto Inc., Tokyo, Japan) were installed on the floor of the laboratory (Figure 1), and a high-speed camera (Noraxon Inc., Scottsdale, AZ, USA) was installed at a distance of 1 m from the pedals, at the same height. TELEmyo DTS (Noraxon Inc.) was used for recording the electromyogram during the braking action. The sampling frequency was 1500 Hz, and the bandpass filter was set at 10–500 Hz. EMG was performed for the following muscles in the right side of the body: the tibialis anterior, soleus, rectus femoris, and biceps femoris. Skin impedance was reduced to <10 kΩ using alcohol-soaked cotton swabs and an abrasive cream (skinPure, Nihon Kohden Co., Ltd., Tokyo, Japan). Ag–AgCl electrodes (EM-272, Noraxon Inc.) were positioned 2 cm apart and were placed at recommended positions by the Surface ElectroMyoGraphy for the Non-Invasive Assessment of Muscle project [18]. Pressure sensors (FSR-406: Interlink Electronics Inc., Camarillo, CA, USA) connected to foot switches (Noraxon Inc.) were attached to the front of the accelerator and brake pedal, and an electronic inclinometer (Noraxon Inc.) was attached to the rear of the accelerator pedal.

MyoMOTION (Noraxon Inc.) was used for recording the kinematics data during the braking action. The sampling frequency of the inertial sensors was set at 100 Hz. MyoMOTION inertial sensors were placed according to the lower limb rigid-body model with seven joint segments used in MR3 software (Noraxon Inc.), as follows: on shoes (top of the upper foot), front of the shanks, front of the thighs, and the bony area of the sacrum [19]. Calibration was performed in an upright position to determine the value of the 0° angle in the joints studied. MyoSync (Noraxon Inc.) was used to synchronize all of the instruments and to ensure alignment of the time frames.

Immediately before the experiment, a medical interview was conducted for the participants to investigate their driving self-confidence (14 levels from −7 to 7 points [20]), history of pedal errors, cognitive function (MMSE), and history of falls within 1 year (Table 1). The posture of the braking action was the sitting position, and the height of the seat or floor was adjusted so that the hip and knee joints were flexed at 90° with the sole on the floor. The chair was completely fixed using a weight to prevent shaking and rotation. The side position of the pedal was adjusted so that the center of the brake was aligned with the midline of the body, and the distance to the front was adjusted so that the subject could apply the brake comfortably. All of the subjects wore the same model of shoes (CSS-01N, Midori Anzen Co., Ltd., Tokyo, Japan), which matched their foot size. A steering wheel was attached to the front end of the elevating table installed above the pedal, and a display was installed 1 m in front of the eyes. At the top of the display, a three-color LED light that could be turned on via a switch was installed; this switch could be operated by an examiner from a distance.

The subjects were instructed to check the real-time data of the accelerator tilt displayed on the screen while holding the steering wheel, and to keep the accelerator depressed 5° to 10°. Then, the examiner randomly turned on the three-color LED at intervals of 20–30 s. The subjects applied the accelerator when the LED light was blue, braked gently when it was yellow, and braked as quickly as possible (emergency brake) when it was red. The subject was free to decide the position of the foot and how to move it. The experiment was conducted in three intervals of 5 min, yielding a total of three sets of measurements. There were no breaks between sets; only the inertial sensor calibration was performed. Finally, HumacNorm (Computer Sports Medicine Inc., Stoughton, MA, USA) was used to perform the maximal isometric contractions of the knee flexion and extension, ankle dorsiflexion, and plantarflexion for 5 s, and the corresponding electromyograms were measured.

### 2.3. Data Analysis

**Figure 2:**
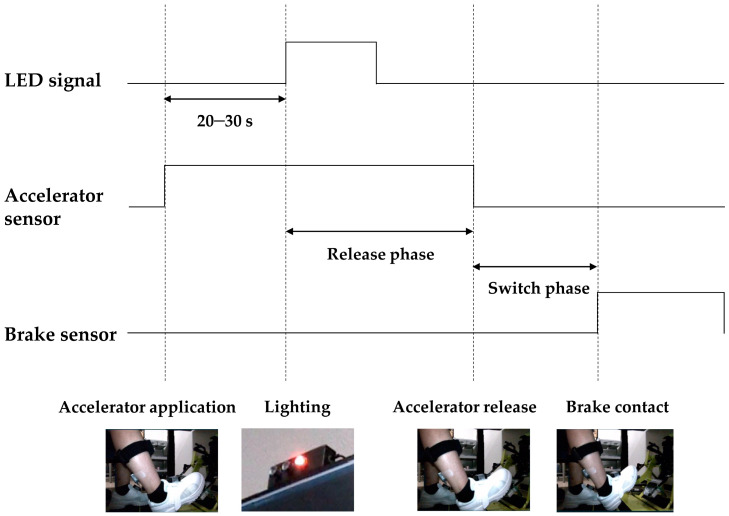
Separation of the phases of the braking action.

MR3 was used for analyzing the kinematic and EMG waveforms. Emergency braking in response to the red LED was analyzed, and the most responsive braking action was extracted from each of the three sets. Based on the potential signals from the LED and pedal pressure sensor, the period from the LED lighting up to the time of reaction of the brake pressure sensor was determined as the analysis section (reaction time). It was divided into the accelerator phase (from 3 s before the LED lighting), the release phase (from LED lighting to release of the accelerator sole), and the switch phase (from the release of the accelerator sole to making contact with the brake; Figure 2). The EMG waveforms were full-wave rectified and smoothened with a low-pass bidirectional second-order Butterworth filter (a cutoff frequency 10 Hz) [21]. Then, the average amplitude during the accelerator application phase, the peak amplitude during the release phase, and the peak and minimum amplitudes of the switch phase were extracted. These values were normalized by dividing them by the peak amplitude during the maximum isometric contraction. The muscle activity onset time was calculated using the mean plus three standard deviations during the accelerator application phase as the threshold level [9,21]. Furthermore, the difference in the activity onset time between the tibialis anterior and soleus was calculated. The duration of co-activation (CoD) was calculated as the ratio of the period when both the flexor and extensor muscles exceeded the onset threshold to the entire period of each phase [21]. The denominator for calculating the release phase CoD was the time from the onset of the flexor or extensor to the release of the accelerator. The CoD of the switch phase was calculated with the time of the entire period as the denominator.

The kinematic patterns of the braking action classified from the high-speed camera images and the skeletal model displayed in MR3 were as follows: (i) fix the heel position and rotate the toes (rotation type), (ii) slide the heel with the toes facing upward (slide type), and (iii) rotate the toes and slide the heel (mixed type). Next, the angles of the right lower limb joint at the accelerator application, accelerator release, maximum value during the switching phase, and brake contact were extracted. The peak joint angular velocities were calculated by differentiating the joint angles of the release and switch phase. Finally, all of the electromyograms and kinematics data were averaged from three braking actions.

### 2.4. Statistical Analysis

Statistical differences in the evaluation data between young and elderly participants were analyzed by the independent *t*-test, Welch’s *t*-test, Mann–Whitney U test, or chi-square test, depending on the data characteristics. Cohen’s d was used to assess the effect size (ES) [22]. For performing these statistical analyses, BellCurve for Excel (Social Survey Research Information Co., Ltd., Tokyo, Japan) was used, with the significance level set at 5%.

## 3. Results

All of the participants passed the eligibility criteria, with an average age of 26.3 ± 5.4 years for young people and 78.4 ± 4.0 years for elderly people (Table 1). There were no significant differences between the groups in height, weight, and shoe size. The self-confidence of driving in the elderly was significantly lower than that in the young (*p* = 0.033; Z = 2.136). The MMSE of the elderly was significantly lower than that of the young (*p* = 0.002; 95% CI: 0.813–3.187), but it was within the normal range in all of the cases. Two young drivers and one elderly driver had a history of pedal error, and one elderly person had a history of falls. All of the subjects performed about 15 emergency brakes in three sets of 5 min duration. For the average reaction time of three braking actions, the elderly drivers were significantly delayed over the entire phase (*p* < 0.001; 95% CI: 0.061–0.163) and the switch phase (*p* = 0.007; 95% CI: 0.027–0.152) compared with the young, but there was no significant difference in the release phase (Table 2).

### 3.1. Electromyographic Activity

**Figure 3:**
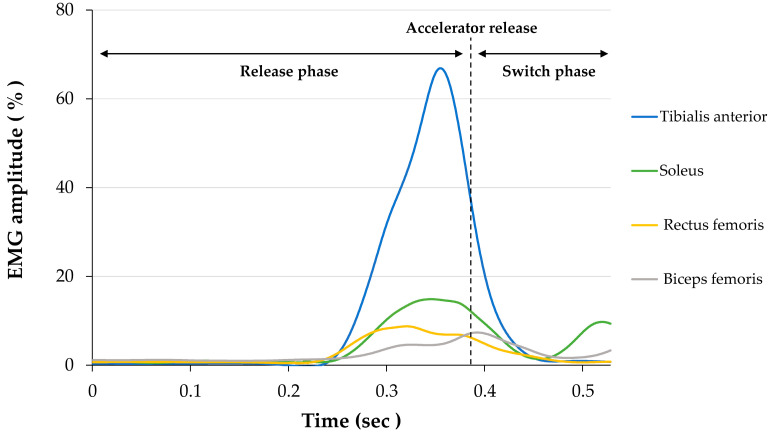
Representative case of the normalized EMG waveforms.

Figure 3 shows the normalized EMG waveforms in one typical young case. In the elderly drivers, the onset of the rectus femoris activity was significantly delayed compared with that in the young drivers (*p* = 0.014; 95% CI: 0.037–0.276), and the onset time difference between the tibialis anterior and soleus was significantly shorter (*p* = 0.033; 95% CI: 0.002–0.043) (Table 2). In the release phase, the amplitude of the tibialis anterior (*p* = 0.028; 95% CI: 0.025–0.379) and soleus (*p* = 0.003; 95% CI: 0.062–0.243) were significantly higher in the elderly drivers. In the switch phase, the peak (*p* = 0.009; 95% CI: 0.074–0.451) and minimum (*p* = 0.001; 95% CI: 0.042–0.129) amplitude of the soleus were significantly higher in the elderly drivers (Table 3). The CoD of the ankle was significantly higher in the elderly drivers during the release phase (*p* = 0.038; 95% CI: 0.006–0.200). The CoD of the knee was significantly lower in the elderly drivers during the release (*p* = 0.028; 95% CI: 0.032–0.484) and switch phases (*p* = 0.041; 95% CI: 0.011–0.463; Table 3).

### 3.2. Kinematics

**Figure 4:**
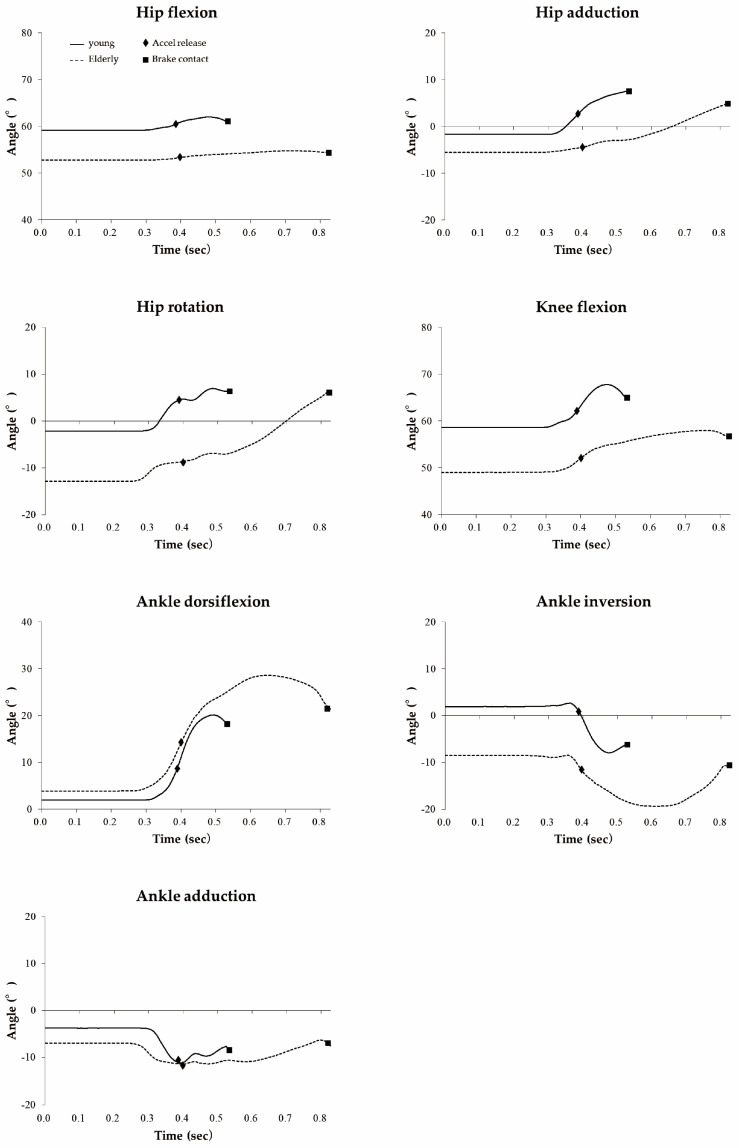
Kinematic data of one case of braking action in each group.

With regard to the movement pattern of the braking action, the ratio of types was not significantly different between the groups (*p* = 0.561; χ^2^ = 1.155; rotation type: young drivers 3 and elderly drivers 5; slide type: young drivers 3 and elderly drivers 2; mixed type: young 5 and elderly 3). Figure 4 shows the joint movements of one typical case in each group. The hip-joint internal rotation angle in the elderly was significantly larger than that in the young drivers during accelerator application (*p* = 0.037; 95% CI: 0.494–14.331), the peak value of the switch phase (*p* = 0.012; 95% CI: 2.866–20.494), and that at the brake contact (*p* = 0.007; 95% CI: 3.821–21.248; Table 4). The knee flexion angle in the elderly drivers was significantly smaller at accelerator application (*p* = 0.018; 95% CI: 2.616–24.249), accelerator release (*p* = 0.020; 95% CI: 2.308–23.471), the peak value during the switch phase (*p* = 0.010; 95% CI: 3.709–24.095), and at brake contact (*p* = 0.009; 95% CI: 4.283–25.620). The ankle adduction angle in the elderly was significantly smaller during accelerator application (*p* = 0.001; 95% CI: 3.017–10.441), at accelerator release (*p* = 0.005; 95% CI: 2.114–10.100), the peak value during switch phase (*p* = 0.044; 95% CI: 0.151–10.194), and at the brake contact (*p* = 0.044; 95% CI: 0.151–10.194). The peak angular velocity of hip adduction in the elderly was significantly lower in the release phase (*p* = 0.039; 95% CI: 1.293–43.174) and the switch phase (*p* = 0.036; 95% CI: 1.486–39.115; Table 5).

## 4. Discussion

According to the previous study [23] conducted using the same method as used in this study, the brake reaction time was about 0.70–0.75 s when there was almost no uncertainty, and the results were almost the same for the young people in this study. The reaction time of the elderly drivers in this study was about 0.1 s longer than that of the young drivers, and the difference was statistically significant. However, there was no difference in the time from LED lighting to the release of the accelerator (release phase) between the young and elderly drivers. The elderly drivers tended to be slower at recognizing dangerous situations owing to the influence of visibility and cognitive load [23], but as simple stimuli were used in this study, the effects of visual acuity and cognitive function were considered to be small. In particular, the brake reaction time in this study was greatly affected by the physical function. An important finding of this study is that the time taken for the foot to be released from the accelerator to the foot making contact with the brake (switch phase) was clearly longer in the case of the elderly drivers.

In previous studies [9,12], the timing of the onset of the tibialis anterior activity was the earliest in the braking action of young drivers, and similar results were obtained in this study, and the amplitude of the tibialis anterior was the highest among the measured muscles. From the release phase to the switch phase, the change in the angle and the angular velocity of the ankle dorsiflexion were larger than those of other joints, and it is clear that the tibialis anterior is the main action muscle involved in the braking action. The soleus, which is the antagonist of the tibialis anterior, started its activity slightly later than the tibialis anterior. The soleus muscle was involved in the action of depressing the accelerator (ankle plantar flexion), but an increase in amplitude was confirmed in the release phase, and the amplitude further increased in the switch phase. In the experimental pedal used in this study, the brake was higher than the accelerator in terms of the height from the floor, as in a general vehicle. Therefore, the ankle joint needed to further increase the dorsiflexion angle and angular velocity after the sole was released from the accelerator. As the muscles are also activated by rapid stretch stimulation [24], the soleus may have induced the stretch reflex because of the increase in ankle dorsiflexion associated with pedal switching. The most important finding in this study was that the soleus amplitude in the elderly was larger than that in the younger participants from the release to the switch phase. Furthermore, the difference in the time of activity onset between the tibialis anterior and soleus in the elderly was less and the co-activity was high compared with those in the younger group. These results indicate that the soleus activity is not suppressed in the elderly.

Normally, when the tibialis anterior is active, the activity of the soleus, which is an antagonist muscle, is suppressed via Ia inhibitory interneurons in the spinal cord [25]. Among the young participants, the onset of soleus activity was later and the simultaneous activity was lower compared with those of the elderly participants during the release phase. The stretch stimulation by ankle dorsiflexion caused the soleus activity, but it is considered that Ia reciprocal inhibition from the tibialis anterior [13,25] was sufficiently effective in the young participants. However, the age-related decrease in reciprocal inhibition may be associated with changes in the transmission efficiency at the Ia afferent terminal on the Ia inhibitory interneurons and (or) the terminal of the Ia interneurons on motor neurons, owing to increased presynaptic inhibition [14,26]. Therefore, in the braking action of the elderly, the ankle dorsiflexion may be inhibited by the increased activity of the soleus. In this study, the tibialis anterior activity in the elderly was high, possibly countering the inhibition of dorsiflexion by the soleus. Hence, the angle and angular velocity of the ankle dorsiflexion were not significantly different between the elderly and the young. In particular, it is unlikely that the increased activity of the soleus muscle affected the time delay of the switch phase. However, it has been reported that elderly drivers exhibit muscle fatigue in the tibialis anterior after driving for 1 h [10]. When the activity of the tibialis anterior is reduced by long-term driving, the ankle joint may be susceptible to the plantarflexion moment (i.e., depressing the accelerator) by the soleus during the release phase. In addition, the movement of the ankle inversion was observed in the switch phase. The main action muscle of the ankle inversion is also the tibialis anterior [27]. When the dorsiflexion of the ankle joint is inhibited by decreased reciprocity inhibition and (or) tibialis anterior fatigue, serious pedal errors such as involuntary accelerator depression and back-pedal hooks may occur.

The onset activity of the rectus femoris occurred slightly later than that of the tibialis anterior in the young drivers, and the electromyographic amplitude was approximately 10%. The rectus femoris has a flexing action on the hip joint [27]. This may reduce the friction between the sole and the floor, making it easier to slide the foot from the accelerator to the brakes. However, the onset of rectus femoris activity in the elderly drivers was significantly later than that in the young drivers. Furthermore, fast fiber (Type II) accounts for about 70% of the muscle fiber type of the rectus femoris muscle, [28], and the ratio is outstandingly large among the test muscles targeted in this study. In view of the skeletal muscle atrophy of type II fibers with aging [29], the onset of rectus femoris muscle activity is considered to be delayed in the elderly. This delay may have affected the movement of the foot on the coronal and the horizontal planes. The angular velocity of hip adduction in the elderly was lower than that in the young. On the contrary, during the switch phase, the hip-joint internal rotation angle was larger in the elderly than in the young. That is, elderly people tended to slide the foot from the accelerator toward the brake slowly because of hip adduction, and they tended to move the foot onto the brake pedal by increasing the hip-joint internal rotation angle. This may be because hip adduction was inhibited because the friction between the sole and floor could not be reduced by hip flexion because of the delay in the onset of the rectus femoris activity. Although the simultaneous activity of the flexor and extensor muscles was related to motor control [20], the CoD between the rectus femoris and biceps femoris in the elderly drivers was significantly lower than that in the young drivers. Therefore, the lack of responsiveness and motor control of the muscles that act during the hip and knee movements in the elderly can cause delays and errors when braking.

In reality, the adductor magnus and gracilis are involved in hip adduction, and the gluteus minimus and tensor fasciae latae are involved in hip internal rotation [27], but these were not regarded as test muscles in this study. This is one limitation of this study. In addition, the activity of the biceps femoris was small in both groups throughout the braking action. The biceps femoris is involved in the flexion and external rotation of the knee joint [27], but when switching pedals, the toes rotate to the left, so there is a high possibility that the right knee undergoes internal rotation. Therefore, it is considered that the activity of the semitendinosus and gracilis, which are also involved in the knee internal rotation [27], is important for the braking action. However, these muscles were also not considered as test muscles in this study. The knee joints of the elderly drivers were more extended than those of the younger drivers throughout the braking action. This indicates that the seat position from the accelerator was farther away in the case of the elderly drivers than in the case of the younger drivers. In this study, subjects were able to brake in a comfortable position with the same foot movements as usual. This prevented unfamiliar situations from affecting the results, but the results were affected by individual differences in foot position and movement method. The sample size was relatively small in this study. However, the conclusions were scientifically drawn using appropriate statistical methods, and reliability is guaranteed. There was a significant difference in MMSE between the groups. However, as the median of the elderly drivers was 28, cognitive function was unlikely to influence the results of this study.

## 5. Conclusions

Elderly drivers took the same amount of time from viewing the visual stimulus to releasing the accelerator pedal as the younger drivers did. However, the elderly drivers took longer to switch to the brake pedal. The soleus of the elderly drivers was highly active and may have interfered with the ankle dorsiflexion associated with pedal switching. As the onset of the rectus femoris activity was delayed and the simultaneous activity between the rectus femoris and biceps femoris was low, the hip and knee joints may lack responsiveness and motor control. Furthermore, elderly drivers tended to have a low hip adduction velocity and tended to switch pedals through hip internal rotation. Alteration in the joint movements and muscle activity of elderly drivers can reduce the operability of pedals and may be related to the occurrence of pedal errors. However, note that the trends of the data obtained in this study may change depending on the vehicle type and driving posture.

## Figures and Tables

**Table 1:** Characteristics of the subjects.

		Young (*n* = 11)	Elderly (*n* = 10)	*p*
Age	(Years)	26.3 ± 5.4	78.4 ± 4.0	<0.001
Sex	(Female/Male)	6/5	5/5	NS
Height	(cm)	164.7 ± 8.8	157.6 ± 6.5	NS
Weight	(kg)	55.4 ± 9.1	56.0 ± 6.5	NS
Shoe size	(cm)	24.5 ± 1.2	24.6 ± 1.1	NS
Driving self-confidence		4 (4.5)	0 (1.5)	0.033
MMSE		30 (0)	28 (2.5)	0.002
Pedal error history	(Yes/No)	2/9	1/9	NS
Fall history	(Yes/No)	0/11	1/9	NS

Values are presented as mean ± standard deviation or median (quartile deviation). MMSE—mini-mental state examination; NS—no significant difference.

**Table 2:** Times of reaction and muscle activity onset during emergency braking.

	Young (*n* = 11)	Elderly (*n* = 10)	*p*	ES
Reaction time (s)				
Entire phase	0.650 ± 0.047	0.762 ± 0.059	<0.001	2.104
Release phase	0.450 ± 0.038	0.473 ± 0.064	NS	0.430
Switch phase	0.199 ± 0.031	0.289 ± 0.088	0.014	1.386
Muscle activity onset (s)				
Tibialis anterior	0.290 ± 0.050	0.320 ± 0.072	NS	0.473
Soleus	0.332 ± 0.047	0.339 ± 0.076	NS	0.108
Rectus femoris	0.316 ± 0.046	0.472 ± 0.156	0.015	1.387
Biceps femoris	0.365 ± 0.036	0.409 ± 0.068	NS	0.823
Time difference between TA and Sol	0.042 ± 0.028	0.019 ± 0.011	0.033	1.052

Values are presented as mean ± standard deviation. ES—effect size; TA—tibialis anterior; Sol—soleus; NS—no significant difference.

**Table 3:** Electromyography amplitude and co-activation duration during emergency braking.

		Young (*n* = 11)	Elderly (*n* = 10)	*p*	ES
Amplitude (%)					
Tibialis anterior	A ^a^	2.3 ± 2.4	3.5 ± 1.9	NS	0.535
	R ^p^	52.6 ± 16.6	72.7 ± 20.2	0.028	1.096
	S ^p^	54.0 ± 21.7	75.9 ± 29.3	NS	0.854
	S ^m^	4.9 ± 4.7	6.7 ± 5.4	NS	0.356
Soleus	A ^a^	2.2 ± 1.3	3.2 ± 1.5	NS	0.686
	R ^p^	12.7 ± 5.7	28.0 ± 11.3	0.003	1.727
	S ^p^	28.4 ± 15.0	55.0 ± 23.6	0.009	1.341
	S ^m^	3.0 ± 1.7	11.5 ± 5.6	0.001	2.086
Rectus femoris	A ^a^	2.3 ± 2.8	3.3 ± 1.5	NS	0.416
	R ^p^	11.6 ± 5.9	10.0 ± 7.3	NS	0.252
	S ^p^	12.0 ± 5.3	13.0 ± 8.7	NS	0.132
	S ^m^	3.4 ± 2.0	3.1 ± 1.2	NS	0.134
Biceps femoris	A ^a^	1.7 ± 1.9	1.5 ± 0.7	NS	0.138
	R ^p^	3.5 ± 2.8	6.4 ± 5.7	NS	0.661
	S ^p^	5.5 ± 3.9	9.6 ± 5.9	NS	0.820
	S ^m^	2.3 ± 2.0	2.8 ± 2.1	NS	0.254
Co-activation duration (%)					
TA―Sol	R	75.8 ± 12.4	86.1 ± 6.5	0.038	1.025
	S	78.9 ± 13.6	89.6 ± 13.5	NS	0.785
RF―BF	R	61.3 ± 23.3	35.5 ± 23.7	0.028	1.097
	S	73.0 ± 22.4	49.3 ± 24.7	0.041	1.009

Values are presented as mean ± standard deviation. A—acceleration application phase; R—release phase; S—switch phase; ^a^—average value; ^p^—peak value; ^m^—minimum value; TA—tibialis anterior; Sol—soleus; RF—rectus femoris; BF—biceps femoris; ES—effect size; NS—no significant difference.

**Table 4:** Joint angle during emergency braking.

		Young (*n* = 11)	Elderly (*n* = 10)	*p*	ES
Hip flexion	A	49.2 ± 12.7	56.3 ± 10.3	NS	0.620
	R	49.7 ± 12.3	56.8 ± 10.8	NS	0.611
	S ^p^	53.5 ± 11.7	59.4 ± 11.0	NS	0.524
	B	52.8 ± 11.7	58.2 ± 11.5	NS	0.460
Hip adduction	A	−2.4 ± 1.7	−3.4 ± 3.3	NS	0.395
	R	−0.1 ± 2.3	−2.2 ± 3.8	NS	0.654
	S ^p^	7.0 ± 3.6	5.1 ± 2.3	NS	0.627
	B	6.8 ± 3.6	4.9 ± 2.2	NS	0.651
Hip internal rotation	A	−5.8 ± 8.6	1.6 ± 5.2	0.037	1.030
	R	−4.1 ± 9.2	3.3 ± 5.0	NS	0.941
	S ^p^	3.9 ± 9.3	15.6 ± 9.0	0.012	1.274
	B	1.2 ± 8.6	13.7 ± 9.0	0.007	1.383
Knee flexion	A	61.7 ± 10.4	48.3 ± 9.5	0.018	1.194
	R	63.9 ± 9.7	51.0 ± 12.3	0.020	1.171
	S ^p^	72.6 ± 8.3	58.7 ± 12.6	0.010	1.311
	B	70.0 ± 8.7	55.0 ± 13.2	0.009	1.348
Ankle dorsiflexion	A	5.9 ± 9.4	0.8 ± 5.4	NS	0.661
	R	12.6 ± 5.8	8.6 ± 6.1	NS	0.669
	S ^p^	27.4 ± 5.5	26.2 ± 4.6	NS	0.234
	B	22.6 ± 5.8	17.8 ± 6.4	NS	0.786
Ankle inversion	A	0.8 ± 7.1	0.7 ± 6.5	NS	0.026
	R	0.2 ± 7.5	−1.5 ± 6.0	NS	0.242
	S ^p^	6.6 ± 9.3	6.6 ± 8.4	NS	0.003
	B	4.5 ± 8.7	5.2 ± 7.9	NS	0.087
Ankle adduction	A	−0.9 ± 3.8	−7.7 ± 3.9	0.001	1.743
	R	−3.0 ± 4.2	−9.1 ± 4.1	0.005	1.471
	S ^p^	3.7 ± 4.3	−1.5 ± 6.1	0.044	0.990
	B	3.0 ± 4.2	−2.4 ± 6.0	0.033	1.056

Values are presented as mean ± standard deviation (degree). A—acceleration application; R—at acceleration release; S—during switch phase; ^p^—peak value; B—at brake contact; ES—effect size; NS—no significant difference.

**Table 5:** Maximum angular velocity of joints during emergency braking.

		Young (*n* = 11)	Elderly (*n* = 10)	*p*	ES
Hip flexion	R	28.8 ± 19.4	23.6 ± 13.3	NS	0.308
	S	48.4 ± 16.7	37.9 ± 19.7	NS	0.577
Hip extension	R	8.2 ± 4.7	10.0 ± 15.1	NS	0.166
	S	24.8 ± 10.1	29.0 ± 11.2	NS	0.393
Hip adduction	R	46.9 ± 26.5	24.7 ± 14.9	0.039	1.021
	S	69.2 ± 24.1	48.9 ± 12.8	0.036	1.037
Hip abduction	R	3.0 ± 2.8	1.8 ± 0.8	NS	0.542
	S	0.5 ± 14.6	9.9 ± 14.2	NS	0.656
Hip internal rotation	R	47.5 ± 30.6	52.7 ± 22.1	NS	0.195
	S	111.2 ± 52.9	138.5 ± 64.1	NS	0.467
Hip external rotation	R	17.4 ± 13.1	26.3 ± 16.9	NS	0.596
	S	63.2 ± 45.7	102.8 ± 49.4	NS	0.834
Knee flexion	R	76.4 ± 39.0	75.9 ± 32.2	NS	0.015
	S	110.8 ± 25.6	108.5 ± 34.2	NS	0.078
Knee extension	R	8.9 ± 9.8	16.3 ± 13.9	NS	0.621
	S	86.9 ± 29.6	84.0 ± 21.9	NS	0.109
Ankle dorsiflexion	R	184.6 ± 88.3	179.4 ± 59.6	NS	0.068
	S	235.9 ± 68.4	226.6 ± 51.8	NS	0.152
Ankle plantarflexion	R	2.8 ± 1.9	6.4 ± 7.7	NS	0.644
	S	203.3 ± 75.3	220.5 ± 51.3	NS	0.265
Ankle inversion	R	28.7 ± 28.1	22.6 ± 18.0	NS	0.256
	S	137.0 ± 47.2	151.3 ± 40.4	NS	0.323
Ankle eversion	R	60.8 ± 50.3	79.1 ± 40.2	NS	0.401
	S	150.1 ± 69.0	167.1 ± 67.4	NS	0.249
Ankle adduction	R	16.3 ± 12.3	30.3 ± 19.5	NS	0.865
	S	118.1 ± 40.0	140.1 ± 71.4	NS	0.385
Ankle abduction	R	53.0 ± 39.2	54.9 ± 27.4	NS	0.057
	S	80.6 ± 45.0	114.4 ± 52.7	NS	0.692

Values are presented as mean ± standard deviation (degree/s). R—release phase; S—switch phase; ES—effect size; NS—no significant difference.

## Data Availability

The data presented in this study are available upon request from the corresponding author. The data are not publicly available because of privacy.
